# *Helicobacter pylori* Infection Increases Insulin Resistance and Metabolic Syndrome in Residents Younger than 50 Years Old: A Community-Based Study

**DOI:** 10.1371/journal.pone.0128671

**Published:** 2015-05-28

**Authors:** Li-Wei Chen, Chih-Yi Chien, Kai-Jie Yang, Sheng-Fong Kuo, Chih-Hung Chen, Rong-Nan Chien

**Affiliations:** 1 Department of Gastroenterology and Hepatology, Chang-Gung Memorial Hospital and University, Keelung, Taiwan; 2 Community Medicine Research Center, Chang-Gung Memorial Hospital and University, Keelung, Taiwan; 3 Metabolism and Endocrinology, Chang-Gung Memorial Hospital and University, Keelung, Taiwan; University of Catanzaro Magna Graecia, ITALY

## Abstract

This study aimed to analyze the influence of *H*. *pylori* infection on insulin resistance and metabolic syndrome (MS) by multivariate analysis of a community-based cohort study. From January 2013 to February 2014,811 subjects were enrolled in a community-based cohort study from the northeastern region of Taiwan. All subjects received a demographic survey and blood tests, including an *H*. *pylori* antibody test, liver biochemistry tests, lipid profiles, sugar/insulin levels for Homeostatic model assessment (HOMA-IR index), and measurements of adipokines and inflammatory cytokines. A total of 264 men and 547 women were included in this study. The mean age was 59.2 ± 12.7 years. Subjects seropositive for *H*. *pylori* antibodies exhibited higher rates of hypertension, an increased incidence of a HOMA-IR index > 2.5 and a higher level of tumor necrosis factor-α than those without *H*. *pylori* antibodies. We found a significant difference in the presence of *H*. *pylori* antibodies between subjects with MS and those without MS (76.7% vs. 53.7%, p = 0.007) among subjects < 50 y/o. A HOMA-IR index >2.5, *H*. *pylori* antibody presence and leptin were predictors for MS in subjects < 50 y/o. The estimated odds ratio of MS for a subject with *H*. *pylori* antibodies was 3.717 (95% CI = 1.086–12.719) times that of a subject without *H*. *pylori* antibodies. In addition, no difference in *H*. *pylori* antibody status was detected for MS prediction in subjects that were ≧ 50 y/o (p = 0.861). In conclusion, subjects with *H*. *pylori* antibodies had a higher incidence of a HOMA-IR >2.5 than those without *H pylori* antibodies. For subjects aged < 50 y/o, the *H*. *pylori* antibody was a predictor for MS.

## Introduction

*Helicobacter pylori* (*H*. *pylori*) infection will induce chronic inflammation and immune responses in the stomach [1–3]. Some inflammatory cytokines and adipokines, such as tumor necrosis factor α (TNF-α) and leptin may be involved in this inflammation or immune response [4–9]. Patients with *H*. *pylori* infection have a lower serum fasting leptin level but a higher TNF-α value [7,9]. Past studies have revealed that a leptin deficiency and a high TNF-α level will induce insulin resistance (IR) [10–13]. IR and central obesity are the key mechanisms for developing metabolic syndrome (MS) [14]. The relationships between *H*. *pylori* infection and IR or MS have been reported by several investigations, including two large Japanese population studies and one meta-analysis study [15–20]. However, other investigations have found conflicting results [21,22]. Most studies did not include cytokine/adipokine data and used different MS criteria. The two most popular MS criteria are the National Cholesterol Education Program—Adult Treatment Panel III (NCEP-ATP III) and the International Diabetes Federation (IDF) criteria [23–26]. Because central obesity (visceral obesity) evaluation is an important element in an MS screening study, it is necessary to apply different waist circumference thresholds to different race/ethnicity groups [24,25,27]. The hypothesis of this study is that *H*. *pylori* infection induces inflammatory cytokines and adipokines, which result in IR and MS. The influence of *H*. *pylori* infection on IR and MS was prospectively studied in a community-based screening program using the NCEP-ATP III MS criteria. Serum inflammatory cytokines or adipokines, such as TNF-α, high sensitivity C-reactive proteins (HS-CRP), adiponectin and leptin, were examined for all enrolled subjects.

## Materials and Methods

From August 2013 to February 2014, a community- based cohort study was performed in the Wanli, Ruifang and Anle districts in the north-eastern region of Taiwan. All subjects participated in a demographic survey, physical examination and blood tests. Waist circumference was measured at the midline between the lowest margin of the subcostal rib and the upper margin of the iliac crest. Body mass index (BMI) was measured as weight (kg) divided by height (meters) squared (kg/m^2^). Blood samples were collected from all participants after overnight fasting and were immediately (within four hours after collection) analyzed for complete blood cell count, biochemistry and antibody titers. Additional samples were transferred into a chilled tube, centrifuged immediately (4°C at 3000 rpm for 30 minutes) and stored at -80°C until assays for adiponectin, leptin and TNF-α were performed.

Subjects with systemic diseases, such as diabetes mellitus (DM), hypertension, hyperlipidemia or chronic kidney disease were recorded. A standardized questionnaire was administered to all subjects by a trained team of interviewers. Information obtained via the questionnaire included comprehensive alcohol consumption (amount, duration, and the AUDIT and CAGE questionnaires), smoking and chewing betel nut status, medication history (oral hypoglycemia agents, insulin injection, statins, herbal medicine, hormones, and antibiotics), family history, and physical activity (SF36 health survey),. Telephone calls for the past drug history of *H*. *pylori* eradication survey were performed following the serum IgG anti *H*. *pylori* test. This research was approved by the Institutional Review Board of the Chang-Gung Memorial Hospital (IRB No: 102-2827C, 103-2392C1). Written informed consent was obtained from all subjects before enrollment in this study.

### *H*. *pylori*-specific immunoglobulin G (IgG) antibody

The IgG anti *H*. *pylori* titer was determined with a commercially available enzyme linked immunosorbent assay (Pyloriset Dry, Orion Diagnostica, Espoo, Finland). The rapid latex agglutination method was used to detect the *H*. *pylori* antibody. The local validation of Pyloriset Dry test had been done in the department of clinical pathology of our hospital. The sensitivity and specificity were around 95% and 82% respectively, which were very near the manufacturer’s instruction data (sensitivity 97%, specificity 85% and agreement 92%, respectively).

### Adiponectin and leptin

The assays for adiponectin and leptin employed the quantitative sandwich enzyme immunoassay technique and were performed according to the manufacturer’s instructions (Human Total Adiponectin/Acrp30, BioVendor Research and Diagnostic system, Minneapolis, USA; Human Leptin ELISA, Clinical Range, BioVendor Laboratory Medicine, Karasek, Czech Republic).

### Tumor necrosis factor alpha (TNF-α)

The TNF-α assay used a quantitative sandwich enzyme immunoassay technique and was performed according to the manufacturer’s instructions (Immunite 1000 LKNF1, Siemens Medical Solutions Diagnostics, Llanberis, UK)

### Glycosylated hemoglobin (Hb A1c)

We used boronate affinity and high-performance liquid chromatography (HPLC) to analyze the percent of glycosylated hemoglobin in the blood samples. The detected range was 3.8~18.5% (Premier Hb9210, Trinity Biotech PLC, Kansas City, USA).

### Homeostasis model assessment of insulin resistance (HOMA-IR)

The estimate of IR was determined by the HOMA score, which was calculated by the formula: [fasting plasma insulin (mU/L) × fasting plasma glucose (mmol/L)]/22.5. A high HOMA-IR score denotes low insulin sensitivity and IR [28]. Although there is no a standard normal range of HOMA-IR, the upper cut-off value of HOMA-IR has been proposed between 2.0 and 3.0 in different populations [18, 20, 22, 28, 29]. To avoid using an arbitrary cut-off value, this study applied three cut-off values of HOMA-IR index (2, 2.5 and 3) to evaluate IR.

### Short Form-36 (SF-36)

The Taiwan version SF-36 questionnaire was applied for quality of life survey [30, 31]. SF-36 consists of eight sections with scaled scores, includes sections of vitality, physical functioning, bodily pain, general health perceptions, physical role functioning, emotional role functioning, social role functioning and mental health. Each scale is directly transformed into a 0–100 scale on the assumption that each question carries equal weight. The lower the score means the more disability. The higher the score mean the less disability. Two aggregate summary measures, the physical component summary (PCS) and the mental component summary (MCS) were also analyzed.

### Metabolic syndrome (MS)

According to the NCEP ATP III criteria [23], a race-specific waist circumference threshold was applied to prevent a discrepancy in MS prevalence. The cut-off values for normal waist circumference in men and women were 90 cm and 80 cm, respectively, for Asian people. In this study, the ATP III criteria define MS as the presence of at least three of the following five traits: 1) abdominal obesity, defined as a waist circumference in men and women of ≥90 cm (35 inches) and ≥80 cm (31.5 inches), respectively; 2) serum triglycerides ≥150 mg/dL (1.7 mmol/L) or drug treatment for elevated triglycerides; 3) serum HDL cholesterol <40 mg/dL (1 mmol/L) in men and <50 mg/dL (1.3 mmol/L) in women or drug treatment for low HDL-C; 4) blood pressure ≥130/85 mmHg or drug treatment for elevated blood pressure; or 5) fasting plasma glucose (FPG) ≥100 mg/dL (5.6 mmol/L) or drug treatment for elevated blood glucose.

Serum insulin and fasting blood sugar were used for the analysis of IR (HOMA-IR) in this study. The analysis of HOMA-IR for IR was not suitable for patients with DM or those that took medicine for blood sugar control [28–29, 32]. Although patients undergoing drug treatment for elevated blood sugar were classified as having MS by the NICE ATPIII criteria, patients treated by oral hypoglycemia agents or insulin injections were excluded from our analysis.

### Statistical analysis

Values are expressed as means and standard deviations (SD) for continuous data. Categorical data were analyzed with the Chi-square test or Fisher’s exact test, as appropriate. The Cochran–Mantel–Haenszel (CMH) statistic was used to test the conditional independence of data in 2×2×2 tables. All statistical tests were 2-tailed. A *p*- value of <0.05 was considered statistically significant. The association between metabolic parameters and *H*. *pylori* serostatus was evaluated by the Pearson correlation coefficient and multivariate logistic regression analysis after adjusting for sex as a potential confounder. Statistical analyses were performed using the statistical package SPSS for windows (Version 14.0, Chicago, IL, USA).

## Results

A total of 829subjects (560 women) were initially enrolled. Eighteen subjects were excluded because of a past history of *H*. *pylori* eradication therapy. These 18 subjeccts were all more than 50 years old and had IgG anti *H*. *pylori*. Then 811 subjects were finally included for this study. The mean age was 59.2 years. The demographic data are listed in Table 1. Serum antibodies for *H*. *pylori* (IgG anti *H*. *pylori*) were positive in 62.8% (509/811) of subjects. According to the ATP III criteria, MS was present in 36.6% (297/811) subjects. The percents of subjects exhibited HOMA-IR indexs greater than 2, 2.5 or 3 were 34.0% (276/811), 25.5% (207/811) and 18.9% (153/811) respectively. Subjects were divided into two groups according to *H*. *pylori* antibody serostatus. The demographic data are shown in Table 2. The positivity ratio of the *H*. *pylori* antibody was approximately 60% in every age stratum and did not change significantly with increasing age.

**Table 1:** Characteristics of Study Subjects.

Gender (women/men)	547/264
Mean age	59.2±12.7
Waist abnormality No (%)	402 (49.6)
Hypertension No (%)	478(58.7)
Hyperlipidemia No (%)	731 (87.9)
Hyperglycemia No (%)	394 (48.6)
Anti *H pylori* Ab (+) No (%)	509(62.8)
Metabolic syndrome No (%)	297 (36.6)
HOMA-IR*	2.4±3.3
BMI (kg/m^2^)*	24.9±3.6
Waist (cm)*	83.5±10.0
SBP (mmHg)*	133.4±19.5
DBP (mmHg)*	79.1±12.6
TG (mg/dL)*	127.9±96.8
HDL (mg/dL)*	57.8±15.1
LDL (mg/dL)*	126.0±33.2
VLDL (mg/dl)*	24.8±16.5
TCHOL (mg/dL)*	211.1±38.8
FBG (mg/dL)*	107.4±29.9
HbA1c (%)*	5.94±0.9
Estimated AG (mg/dL)*	123.7±25.8.
Insulin (mU/L)*	8.5±9.8
AST (U/L)*	26.1±15.9
ALT (U/L)*	25.8±21.7
HS-CRP (mg/L)*	1.9±3.4
TNF-α (ng/ml)*	5.9±2.3
Adiponectin (μg/ml)*	7.8±6.0
Leptin (μg/ml)*	13.6±8.7

*: mean±standard deviation (SD)

*H*. *pylori* Ab: *Helicobacter pylori* antibody

HOMA-IR: Homeostasis model assessment of insulin resistance

BMI: body mass index

SBP: systolic blood pressure

DBP: diastolic blood pressure

TG: triglyceride

HDL: high density lipoprotein

LDL: low density lipoprotein

VLDL: very low density lipoprotein

TCHOL: total cholesterol

FBG: fasting blood glucose

HbA1c: glycosylated hemoglobin

AG: average glucose

AST: aspartate aminotransferase

ALT: alanine aminotransferase

HS-CRP: high sensitivity C-reactive protein

TNF-α: tumor necrosis factor α

**Table 2:** Demographic data among patients with or without the *H*. *pylori* antibody.

	*H*. *pylori* Ab (-)	*H*. *pylori* Ab (+)	P-value
Number (women/men)	302 (200/102)	509 (134/162)	0.567
Age (y/o) Mean	59.0±12.8	59.3±12.8	0.770
Stratum 30–39 (%)	30 (39.5)	46 (60.5)	0.677‡
40–49 (%)	43 (41.3)	61 (58.3)	0.677‡
50–59 (%)	71 (33.2)	143 (68.8)	0.677‡
60–69 (%)	89 (39.4)	137 (60.6)	0.677‡
70–79 (%)	55 (35.9)	98 (64.1)	0.677‡
80–89 (%)	14 (35.9)	25 (64.1)	0.677‡
Abnormal waist (%)†	142 (47.0)	260 (51.1)	0.264
Hypertension (%)	164(54.3)	314 (61.7)	0.039*
Hyperlipidemia (%)	257(85.1)	456 (89.6)	0.058
Hyperglycemia (%)	138(45.7)	256 (50.3)	0.205
Metabolic syndrome (%)	101 (33.4)	196 (38.5)	0.148
HOMA-IR index	2.2±3.2	2.6±3.4	0.144
>2 (%)	90 (29.8)	186 (36.5)	0.050*
>2.5 (%)	65 (21.5)	142 (27.9)	0.044*
>3 (%)	46 (15.2)	107 (21.0)	0.042*
BMI (kg/m^2^)	24.6±3.6	25.1±3.6	0.051*
Waist (cm)	83.3±9.7	83.6±10.1	0.649
SBP (mmHg)	131.6±19.4	134.5±19.5	0.047*
DBP (mmHg)	77.3±12.9	80.2±12.2	0.002*
TG (mg/dL)	119.4±86.2	133.0±102.4	0.055
HDL (mg/dL)	57.6±13.8	57.9±15.8	0.751
LDL (mg/dL)	124.5±33.7	126.9±32.9	0.312
VLDL (mg/dl)	23.4±14.6	25.7±17.4	0.063
TCHOL (mg/dL)	207.9±39.1	213.0±38.5	0.073*
FBG (mg/dL)	105.6±25.0	108.5±32.4	0.183
HbA1c (%)	5.9±0.8	6.0±1.0	0.504
Estimated AG (mg/dL)	123.0±23.3	124.2±27.1	0.509
Insulin (mU/L)	7.7±8.8	8.9±10.3	0.085
AST (U/L)	25.7±20.9	26.3±11.9	0.593
ALT (U/L)	24.4±25.4	26.7±19.2	0.134
HS-CRP (mg/L)	2.0±3.7	1.9±3.2	0.591
TNF-α (ng/ml)	5.6±2.4	6.1±2.3	0.008*
Adiponectin (ng/ml)	7.6±6.0	7.9±6.0	0.419
Leptin (ng/ml)	14.1±9.3	13.4±8.4	0.310

† waist circumference abnormal: men >90 cm, women >80 cm

* *p*-value <0.05

‡ the distribution between subjects with *H*. *pylori* antibody and those without this antibody by age stratum

When compared with subjects without the *H*. *pylori* antibody, subjects with the *H*. *pylori* antibody had higher rates of hypertension, HOMR-IR (including index >2, >2.5 or >3), and higher diastolic/ systolic blood pressure, cholesterol and TNF-α. However, there was no significant difference in the mean serum adiponectin, leptin and HS-CRP values between these two groups.

A receiver operating characteristics (ROC) curve was applied to establish the optimal age cut-off point to differentiate the MS distribution between subjects with or without *H*. *pylori* antibodies. The optimal cut-off point was computed by maximization of Youden’s index (sensitivity+specificity-1) in the ROC curve analysis. Table 3 shows the sensitivity, specificity and Youden’s index. We found that Youden’s index was maximal (0.034) at age 50. Hence, the cut-off point was set to 50 y/o to differentiate the MS distribution between subjects with or without *H*. *pylori* antibody.

**Table 3:** Sensitivity, specificity and Youden’s index at different age cut-off points.

Age	sensitivity	1-specificity	Youden’s index
30	1	1	0
40	0.910	0.901	0.009
50	0.792	0.758	0.034
60	0.511	0.523	-0.012
70	0.242	0.228	0.014
80	0	0	0

The *H*. *pylori* infection rate, frequency of MS and HOMA-IR stratified by age are shown in Table 4 and Table 5. Among subjects less than 50 y/o (Table 4), there were significant differences in the rates of *H*. *pylori* antibody seropositivity and HOMA-IR indexs between subjects with MS and those without MS (*H*. *pylori* antibody rate 76.7% vs. 53.7%, *p* = 0.007; HOMA-IR >2 rate, 67.4% vs. 15.4%, >2.5 rate, 60.5% vs. 9.6%, >3 rate, 46.5% vs. 5.1%, all *p*<0.001). In Table 5, the MS rates were significantly different between subjects with the *H*. *pylori* antibody and those without the *H*. *pylori* antibody in different age groups (*p*-value < 0.001).

**Table 4:** Characteristics of metabolic syndrome status between different age groups.

Age (y/o)	<50			≧50		
	MS(-) (N = 136)	MS(+) (N = 43)	*p*-value	MS(-) (N = 378)	MS(+) (N = 254)	*p*-value
Age	40.8±5.9	41.0±5.2	0.866	63.6±8.9	65.4±9.0	0.012
Leptin	11.7±6.4	17.7±7.6	<0.001	11.4±7.6	17.2±10.0	<0.001
Adiponectin	7.3±6.9	4.9±2.6	0.017	9.0±6.4	6.8±5.2	<0.001
TNF-α	5.3±1.8	5.1±1.2	0.656	5.8±2.3	6.4±2.6	0.001
AST	23.5±13.0	27.7±14.9	0.080	25.7±18.3	27.8±13.2	0.101
ALT	23.1±19.3	35.0±24.4	0.001	23.3±21.7	29.5±21.6	<0.001
HS-CRP	1.4±2.3	2.4±2.4	0.014	1.7±3.5	2.5±3.8	0.003
HOMA-IR†						
>2	21(15.4%)	29(67.4%)	<0.001	70(18.5%)	156(61.4%)	<0.001
>2.5	13(9.6%)	26(60.5%)	<0.001	44(11.6%)	124(48.8%)	<0.001
>3	7(5.1%)	20(46.5%)	<0.001	27(7.1%)	99(39.0%)	<0.001
*H*. *pylori* Ab†	73(53.7%)	33(76.7%)	0.007	240(63.5%)	163(64.2%)	0.861

† data expressed as No (%)

**Table 5:** *H*. *pylori* antibody status, proportion of metabolic syndrome and HOMA-IR in subjects greater or less than 50 years of age.

Age (y/o)	<50 (N = 179)	≧50 (N = 632)	*p*-value
Average age	40.9±5.7	64.2±8.9	<0.001
MS rate (%)	43(24.0%)	254(40.2%)	<0.001
*H*. *pylori* Ab (positive %)	106(59.2%)	403(63.8%)	0.267
HOMA-IR			
>2	50(27.9%)	226(35.8%)	0.051
>2.5	39(21.8%)	168(26.6%)	0.194
>3	27(15.1%)	126((19.9%)	0.143
MS rate in *H*. *pylori* Ab (+)	33/106(31.1%)	163/403(40.4%)	<0.001‡
MS rate in *H*. *pylori* Ab (-)	10/73(13.7%)	91/229 (39.7%)	<0.001‡

† MS: metabolic syndrome

‡ *p*-value of Cochran-Mantel-Haenszel test for the independence of age and *H*. *pylori* antibody controlling for the factor of metabolic syndrome.

The *H*. *pylori* antibody was correlated with MS in subjects < 50 y/o (r^2^ = 0.201, p = 0.007), which is shown in Table 6. Our hypothesis suggested that *H*. *pylori* infection induces the expression of inflammatory cytokines (TNF-α), which results in IR and MS. Although the factor TNF-α was correlated with *H*. *pylori* infection in the > 50 y/o group, this finding could not support our hypothesis because there was no statistical correlation between the *H*. *pylori* antibody status and MS in this group. Further investigations are required to determine whether other inflammatory cytokines or adipokines can induce IR and MS.

**Table 6:** Correlations among metabolic syndrome, *H*. *pylori* status and other factors.

	Metabolic syndrome		*H*. *pylori* Ab	
Age (y/o)	<50 (N = 179)	≥50 (N = 632)	<50 (N = 179)	≧50 (N = 632)
Leptin†	0.360 (<0.001)	0.314 (<0.001)	0.126 (0.107)	-0.075 (0.067)
Adiponectin†	-0.181 (0.017)	-0.180 (<0.001)	0.112 (0.158)	0.001 (0.972)
TNF-α†	-0.036 (0.656)	0.134 (0.001)	0.022 (0.790)	0.106 (0.009)
HS-CRP†	0.183 (0.014)	0.116 (0.003)	0.030 (0.689)	-0.013 (0.443)
HOMA-IR†				
>2	0.495 (<0.001)	0.439 (<0.001)	0.111 (0.138)	0.054 (0.174)
>2.5	0.527 (<0.001)	0.413 (<0.001)	0.135 (0.071)	0.051 (0.198)
>3	0.494 (<0.001)	0.391 (<0.001)	0.127 (0.089)	0.055 (0.169)
*H*. *pylori* Ab†	0.201 (0.007)	0.007 (0.862)	1.000	1.000
MS†	1.000	1.000	0.201 (0.007)	0.007 (0.862)

† data expressed as coefficient (*p* value)

A logistic regression analysis including the covariates leptin, adiponectin, TNF-α, AST, ALT, HS-CRP, HOMA-IR (>2, 2.5 or 3) and *H*. *pylori* antibody serostatus was performed. The associations with predictors and the presence of MS are listed in Table 7. A HOMA-IR index >2.5, *H*. *pylori* antibodies and leptin were found to be predictors of MS in subjects less than 50 y/o. The estimated odds of MS for a subject with the *H*. *pylori* antibody was 3.717 (95% CI = 1.086–12.719) times that of a subject without the *H*. *pylori* antibody. In addition, no difference in *H*. *pylori* antibody status was detected for MS prediction in subjects more than 50 y/o (p = 0.462). When we applied the different HOMA-IR cut-off values (2 or 3), the result of *H*. *pylori* antibody as a predictor for MS was the same as using the HOMA-IR cut-off value of 2.5.There were no significant differences for educational districts and occupational status between subjects with *H*. *pylori* antibodies and those without antibodies in this study.

**Table 7:** Predictors of metabolic syndrome by multivariate logistic regression analysis.

Age (y/o)	<50		≧50	
Variables	Exp(B) (95% CI)†	P value	Exp(B) (95% CI)†	p value
Sex	2.431(0.634–9.324)	0.195	1.170(0.751–1.824)	0.487
Leptin	1.142(1.043–1.250)	0.004	1.060(1.034–1.087)	<0.001
Adiponectin	0.869(0.734–1.029)	0.103	0.945(0.910–0.981)	0.003
TNF-α	0.964(0.645–1.439)	0.856	1.133(1.046–1.229)	0.002
AST	0.969(0.920–1.020)	0.233	0.946(0.914–0.979)	0.002
ALT	1.014(0.972–1.058)	0.514	1.049(1.019–1.079)	0.001
HS-CRP	1.011(0.805–1.269)	0.926	1.014(0.958–1.073)	0.632
HOMA-IR(>2.5)	12.683(3.689–43.604)	<0.001	4.559(2.876–7.229)	<0.001
*H*. *pylori* Ab	3.717(1.086–12.719)	0.036	0.857(0.568–1.292)	0.462

†95% CI: 95% confidence interval

Among the SF-36 domain, subjects with MS had lower scores than those without MS only in the sections of physical function (79.7 ± 24.1 vs. 86.4 ± 19.3, p = 0.000), general health perceptions (57.3±17.8 vs. 60.2±17.8, p = 0.033) and physical component summary (51.6 ± 7.8 vs. 53.5 ± 6.6, p = 0.001). There was no statistical difference in the scores of other sections, including vitality, bodily pain, physical role functioning, emotional role functioning, social role functioning, mental health and mental component summary, between the subjects with MS and those without MS. However, there was no statistical difference of the score among any SF-36 section between subjects with and without anti *H*. *pylori* IgG sero-positivity.

## Discussion

According to a systemic review, there is a potential association between *H*. *pylori* infection and IR [20]. Although the mechanism that links *H*. *pylori* infection and IR remains unclear, a chronic inflammatory response and the associated cytokine release following *H*. *pylori* infection may be responsible for IR pathogenesis [18–20]. The prevalence of both *H*. *pylori* infection and MS were higher in aged patients [33, 34]. A coincidence of *H*. *pylori* infection and MS has been reported by some studies [33,35,36], but conflicting results supporting a role of *H*. *pylori* in promoting MS and IR have been reported by other studies [15,18–20,37,38]. According to Gunji et al., in two large Japanese population studies, *H*. *pylori* infection was significantly associated with IR and MS regardless of subject age. However, the mean age of enrolled subjects in their study was 47.3 y/o, which was younger than the mean age of our enrolled subjects (59.2 y/o). The current study revealed that age classification may be important for the analysis of relationships between *H*. *pylori* infection and IR/MS. Among subjects aged < 50 y/o, subjects with an *H*. *pylori* infection may have a higher likelihood of acquiring IR and MS than those without *H*. *pylori* infection. People with the *H*. *pylori* antibody have a nearly 3.7 times higher likelihood of having MS than those without the *H*. *pylori* antibody in this study. However, among subjects aged > 50 y/o, the rate of MS distribution was not significantly different regardless of *H*. *pylori* infection. This may be due to the presence of more risk factors for the development of MS in people > 50 y/o, such as hyperlipidemia, hyperglycemia or hypertension. Other factors, such as systemic or local inflammations and being overweight may affect *H*. *pylori* inflammation and MS development.

Because most patients acquire *H*. *pylori* infections during childhood, the inflammatory influences of *H*. *pylori* infection on MS and IR might begin or show prominence at a young age. Most adult patients who got *H pylori* infection at childhood are asymptomatic. There was no statistical difference in the scores among any SF-36 section between subjects with and without anti *H pylori* IgG sero-positivity in the current study. This finding may imply *H pylori* infection dose not influence quality of life in the most of our subjects. In this study, the seropositive rate of the *H*. *pylori* antibody was consistently approximately 60% in all age classifications (30 to 80 y/o), which indicates that most enrolled patients acquire *H*. *pylori* infections before 30 years of age. Data collection from adolescents or young adults is necessary for future studies to elucidate the role of current *H*. *pylori* infections in MS development. Because the accuracy of serology test for *H pylori* antibody may be lower in elderly subjects [39], elderly subjects with detected serum *H*. pylori antibodies may have no current *H*. *pylori* infection. This condition might partially explain the observation in our study, which showed *H*. *pylori* infection (by serology test) could not be a predictor for MS in aged subjects more than 50 y/o.

Previous studies have shown that the presence of *H*. *pylori* antibodies is associated with an altered serum lipid profile and is considered a risk factor for atherosclerosis [40,41]. It is well known that TG levels rise and HDL cholesterol levels decline post *H*. *pylori* infection [40–42]. The interaction between *H*. *pylori* and lipid levels may be mediated by adipokines, such as adiponectin and leptin, or inflammatory cytokines, such as HS-CRP or TNF-α [4–11]. These cytokines affect metabolic risk factors, including blood pressure, lipid profile, glucose intolerance, atherosclerosis and cardiovascular diseases [10–14]. In the current study, factors such as adiponectin, leptin, HS-CRP or TNF-α were highly associated with MS in the correlation and regression analysis, which is compatible with previous studies [4–11]. Higher serum mean TG, VLDL, and cholesterol values and increased blood pressure were observed in the subjects with *H*. *pylori* antibodies compared to those without *H pylori* antibodies.

When multivariate logistic regression analysis was applied, with factors including adiponectin, leptin, TNF-α, HS-CRP, AST, ALT, HOMA-IR index and *H*. *pylori* antibody status, *H*. *pylori* antibody status was found to be a predictor for MS in subjects aged < 50 y/o. A possible reason for the correlation between *H*. *pylori* antibody status and MS was IR. The factor of HOMA-IR was also correlated with *H*. *pylori* antibody status in this study. However, there was no significant difference in the mean serum values of adiponectin, leptin and HS-CRP between subjects with *H*. *pylori* antibodies and those without *H*. *pylori* antibodies. Only the TNF-α mean value was higher in subjects with *H*. *pylori* antibodies than those without antibodies. Other cytokines or adipokines in addition to adiponectin, leptin, TNF-α or HS-CRP may be involved in the interaction between IR and *H*. *pylori* infection. Hence, other cytokines or adipokines, such as interleukin 6 or 8, ghrelin or resistin may be utilized to elucidate the pathogenic mechanisms of MS and *H*. *pylori* infection in the future.

Some limitations were present in this study. First, *H*. *pylori* infection status was evaluated solely with an *H*. *pylori*-specific IgG antibody without other confirmed assessments such as a urease breath test or a rapid urease test. The presence of the *H*. *pylori* antibody does not completely correspond to current *H*. *pylori* infection [37]. However, the serostatus of the *H*. *pylori* antibody maybe accurate for determining current *H*. *pylori* infection in young patients. Because most patients acquire *H*. *pylori* infection during childhood and maintain chronic infection status for a long time, the serostatus of *H*. *pylori* antibody may remain constant in young patients if they do not receive *H pylori* eradication therapy. Moreover, the serology test for the *H*. *pylori* antibody is a highly sensitive and cheap mass screening method that can be used in areas with a high prevalence *H*. *pylori* infection, such as the areas examined in the present study.

The second limitation was selection bias. Our study originated from community-based health checkup data. The participants may consist of older adults with underlying diseases and a desire for medical examinations. There was only 179 subjects aged less than 50 years old included in this study. As with the other cross-sectional study designs, unmeasured confounding factors or covariates might exist in this study.

In conclusion, subjects with *H*. *pylori* antibodies had a higher prevalence of IR (HOMA-IR >2), hypertension, and increased TNF-α than those without *H pylori* antibodies. For subjects < 50 y/o, seropositive status for the *H*. *pylori* antibody was a predictor for MS. The estimated odds of MS in a subject with *H*. *pylori* antibodies was 3.717 (95% CI = 1.086–12.719) times higher than that of a subject without *H*. *pylori* antibodies when a cut-off value of 2.5 for HOMA-IR index. However, further study is required to determine if a causal relationship exists between *H*. *pylori* and MS.
